# An Unforeseen Diagnosis After Liver Transplantation for Acute Liver Failure: Extranodal NK/T‐Cell Lymphoma

**DOI:** 10.1155/crhe/9927354

**Published:** 2026-01-14

**Authors:** Giulia L. Soares, Beatriz B. V. Weffort, Lucas V. S. Makarausky, Mariana F. Juste, Renata Z. Silva, Samira A. C. Leila, Wysterlanyo K. P. Barros, Amanda C. Saraiva, Guilherme E. Felga, Jackson A. Barbosa, Nelson Hamerschlak, Marcelo B. de Rezende, Vanderlei Segatelli

**Affiliations:** ^1^ Liver Unit, Hospital Israelita Albert Einstein, São Paulo, State of São Paulo, Brazil, einstein.br; ^2^ Hematology Department, Hospital Israelita Albert Einstein, São Paulo, State of São Paulo, Brazil, einstein.br; ^3^ Pathology Department, Hospital Israelita Albert Einstein, São Paulo, State of São Paulo, Brazil, einstein.br

## Abstract

Acute liver failure (ALF) is a medical emergency characterized by hepatic encephalopathy in patients with recent‐onset jaundice and coagulopathy. We present a case of a patient who developed ALF secondary to NK/T‐cell lymphoma, with the diagnosis confirmed via histopathological analysis of the explanted after liver transplantation. A 50‐year‐old woman with no significant medical history was transferred to our institution with severe acute liver injury. On admission, she exhibited drowsiness, jaundice, and hepatosplenomegaly. The patient denied alcohol use but reported consuming horsetail tea (*Equisetum* spp). We hypothesized drug‐induced liver injury as the main diagnosis, attributing to the use of horsetail tea. Despite intensive supportive care, her condition continued to deteriorate, prompting urgent deceased‐donor liver transplantation. Histopathological analysis of the explant revealed hepatic parenchyma infiltration by atypical lymphoid cells positive for CD2, CD3, CD8, CD56, and CD38, with Ki‐67 > 95% and positive EBV in situ hybridization. The graft biopsy and appendix showed identical findings, confirming a high‐grade Stage IV extranodal NK/T‐cell lymphoma (ENKTL). Given her critical condition, a modified chemotherapy regimen was initiated. Subsequent complications included severe chemotherapy‐induced pancytopenia, febrile neutropenia, invasive aspergillosis, and multiorgan failure, culminating in death 33 days post‐transplant. This case highlights the diagnostic challenges of rare non‐Hodgkin lymphoma with extranodal presentations. Although horsetail tea is associated with liver injury, its contribution to ALF remains insufficiently defined, reinforcing the importance of excluding alternative causes. ENKTL and other malignancies should be considered in indeterminate ALF, even without imaging findings, atypical lymphocytes in peripheral blood/ascites, or overt clinical suspicion of malignancy.

## 1. Introduction

Severe acute liver injury (ALI) is a syndrome characterized by liver damage with impaired function, in a patient without chronic liver disease. Acute liver failure (ALF) may develop as a progression of ALI and is a medical emergency defined by hepatic encephalopathy in patients with recent‐onset jaundice and coagulopathy. It typically requires specialized intensive care and often urgent liver transplantation. Identifying the underlying etiology of ALF is critical, as it may guide bridge therapies to transplantation or, in some cases, eliminate the need for transplantation through targeted treatments. Diagnosing ALF secondary to neoplastic infiltration of the hepatic parenchyma is particularly challenging. We present a case of a patient who developed ALF initially attributed to herb‐induced liver injury (HILI) due to horsetail tea but was ultimately determined to be secondary to natural killer (NK)/T‐cell lymphoma, with the diagnosis confirmed via histopathological analysis of the explant after liver transplantation.

## 2. Case Report

A 50‐year‐old woman with no significant medical history presented a 2‐week history of fatigue, nausea, and vomiting. She was transferred to our institution with severe ALI. On hospital admission, she manifested drowsiness, jaundice, and hepatosplenomegaly. Notable laboratory findings are described in Table 1, corresponding to a MELD‐Na score of 27. Imaging studies revealed heterogeneous hepatomegaly, medius splenomegaly, moderate amount of free fluid in the peritoneal cavity, and Doppler study of the portal system within normal limits. There were no signs of neurological compromise at clinical presentation. Serologies for hepatotropic and nonhepatotropic viruses, autoantibodies panels, Alpha‐1‐antitrypsin, ceruloplasmin, and infectious screening were negative. The patient denied alcohol use but reported consuming horsetail tea (*Equisetum spp.*) some days before the initial symptoms. Liver enzymes continued to rise, and plasmapheresis was indicated, due to progressive laboratory deterioration and increase in MELD‐Na to 30. After plasmapheresis, a diagnostic paracentesis was performed, revealing a serum‐ascites albumin gradient consistent with portal hypertension but no other significant findings. Vasoactive agents were not required up until this moment.

**Table 1:** Laboratory findings at hospital admission.

Laboratory findings at hospital admission	Result	Reference range
Hemoglobin (mg/dL)	10.1	12–15.5
Hematocrit (%)	28.2	35–45
Mean corpuscular volume (fL)	83.4	82–98
Mean corpuscular hemoglobin (pg)	35.8	26–34
Red cell distribution width (%)	17.2	11.5–16.5
Leukocytes (mm^3^)	6410	3500–10,500
Segmented neutrophils (mm^3^)	3397	1700–8000
Lymphocytes (mm^3^)	1154	900–2900
Platelets (μL)	110,000	150,000–450,000
INR	2.03	0.96–1.30
AST (U/L)	2084	< 32
ALT (U/L)	1336	10–35
ALP (U/L)	744	35–104
GGT (U/L)	753	5–36
Total bilirubin (mg/dL)	21.4	< 1.2
Direct bilirubin (mg/dL)	19.8	< 0.3

*Note:* AST: aspartate aminotransferase; ALT: alanine aminotransferase; ALP: alkaline phosphatase; GGT: gamma‐glutamyltransferase.

Abbreviation: INR, international normalized ratio.

A transjugular liver biopsy was performed to further refine differential diagnosis, which revealed histologic features consistent with acute lobular hepatitis. The biopsy specimen included areas with sparse lymphoid cells; however, it did not contain a sufficiently dense lymphoid infiltrate to warrant additional immunohistochemical staining or further characterization of the lymphocytes. Based on this information, HILI secondary to horsetail tea was the main diagnostic hypothesis. Despite intensive supportive care, there was no clinical or laboratory improvement. Her condition continued to deteriorate by progressing to ALF and prompting urgent deceased‐donor liver transplantation. The liver transplantation occurred twelve days after hospital admission, with no intraoperative complications.

The postoperative course was marked by persistent cholestasis, suggestive of biliary stenosis, leading to an endoscopic retrograde cholangiopancreatography. On Postoperative Day 11, she developed a perforated acute abdomen requiring exploratory laparotomy to perform repairs. A tactical appendectomy and liver graft biopsy were also conducted. Histopathological analysis of the explant revealed hepatic parenchyma infiltration by atypical lymphoid cells positive for CD2, CD3, CD8, CD56, and CD38, with Ki‐67 > 95% and positive Epstein–Barr virus (EBV) in situ hybridization. The graft biopsy and appendix showed identical findings, supporting the diagnosis of a high‐grade Stage IV extranodal NK/T‐cell lymphoma (ENKTL) (Figures 1(a), 1(b), and 1(c)). Due to clinical impairment, a modified chemotherapy regimen with cyclophosphamide, doxorubicin, and prednisone was initiated. Vincristine was omitted due to metabolic ileus, doxorubicin reduced by 75%, and cyclophosphamide by 25%, due to hepatic impairment. The patient showed no clinical improvement despite treatment, and subsequent complications included severe chemotherapy‐induced pancytopenia, febrile neutropenia, invasive aspergillosis, septic shock, culminating in multiorgan failure, and death 33 days post‐transplant. No written consent has been obtained from the patients as there are no patient identifiable data included in this case report.

Figure 1Explant liver biopsy. (a) Neoplastic cells show focal and diffuse involvement of the liver parenchyma (hematoxylin and eosin, 20x and 200x). (b) *In situ* hybridization reveals EBV‐positive neoplastic cells (Epstein–Barr virus–encoded small RNAs, 20x). (c) Immunohistochemistry shows neoplastic cells positive for CD3 (left) and CD56 (right) (200x).

**Figure:**
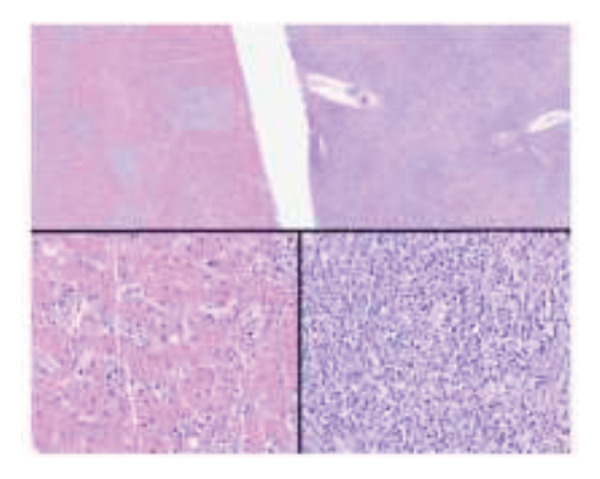
(a)

**Figure:**
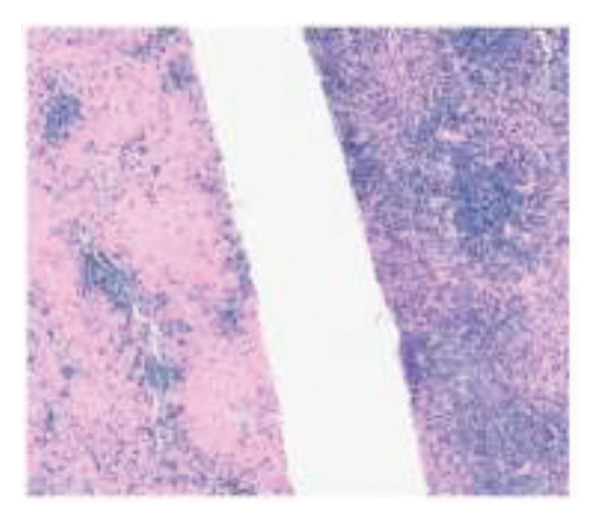
(b)

**Figure:**
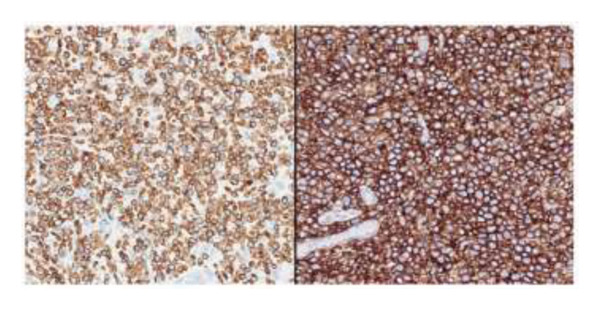
(c)

## 3. Discussion

This case of a 50‐year‐old woman with ALF initially attributed to horsetail tea underscores the diagnostic complexity of rare ALF etiologies. Extensive investigation must rule out viral, autoimmune, vascular, metabolic, and ischemic causes, but malignancy is sometimes overlooked.

T‐ and NK‐cell are non‐Hodgkin lymphomas (NHLs), derived from immature T‐cells or, more commonly from mature T‐ and NK‐cells. More than 30 neoplastic entities are recognized by the International Consensus Classification and by the World Health Organization [1]. ENKTL is an aggressive type of NK/T‐cell lymphoma associated with EBV infection, having an intermediate to a poor prognosis according to staging [2]. Although it most frequently involves the nasal cavity, it can also present at extranasal sites, such as the gastrointestinal tract, lung, and liver. ALF secondary to NK/T‐cell lymphoma is very uncommon, with only two prior cases reported [3, 4]. Both patients presented with jaundice, encephalopathy, and ascites, with lymphomatous infiltration confirmed only via liver biopsy or explant. As noted by Rajvanshi et al., hepatic involvement in lymphomas is typically asymptomatic, manifesting as isolated ALP elevation. Additionally, ALF due to neoplastic infiltration is rare, and imaging modalities such as computed tomography may lack diagnostic utility in infiltrative patterns mimicking viral or drug‐induced hepatitis [5].

The American College of Gastroenterology recommends liver biopsy for ALF with suspected infiltrative or autoimmune disease. However, evidence supporting its diagnostic superiority over clinical assessment alone remains limited [6]. Histologic findings in NK/T‐cell lymphomas may be nonspecific without clinical correlation [7]. Differently from the previous case reports, our patient did not present initially with encephalopathy or ascites. The initial biopsy showed no evidence of malignancy, which made horsetail tea the most plausible explanation for the injury. A systematic review and meta‐analysis of 446 case reports found that HILI is normally associated with a good prognosis, once the toxic product is withdrawn. Horsetail tea use was described only in 4.4% of reported cases, and the most common clinical presentation was jaundice (46.3%). Only 6.6% of all HILI’s cases deteriorated into ALF and required liver transplantation. Concerning liver injury tests, ALP was overall elevated two times its respective upper limit of normality [8]. Moreover, even though our patient ceased exposure to potentially hepatotoxic herbal products weeks before, there was no clinical or biochemical improvement.

On the other hand, hepatic infiltration in lymphomas is found in 16%–27% of NHL patients via percutaneous biopsy and 52%–56% via laparotomy or autopsy [9]. Thompson et al. described four cases of lymphoma‐induced ALF referred for liver transplantation. All patients had ALF symptoms without initial lymphadenopathy, though hepatomegaly was universal. One patient achieved remission with chemotherapy. B‐cell NHL was more frequent (3/4 cases) than Hodgkin lymphoma. In one case, transjugular biopsy initially showed only inflammatory cells without immunohistochemistry; post‐transplant explant analysis revealed malignancy, similarly to our patient [10]. The diagnosis was confirmed only post‐transplant, with a diffuse lymphomatous infiltration identified in the explanted native liver, graft, and appendix, supporting the causality of the aggressive neoplastic disease and the liver failure. As this was such a rapid progression of a high‐grade NHL, a new diagnostic interpretation was made that the initial presentation of ALF occurred due to neoplastic infiltration.

This highlights the diagnostic challenges of rare NHL with extranodal presentations. Although horsetail tea is associated with liver injury, its contribution to ALF remains insufficiently defined, reinforcing the importance of excluding alternative causes. ENKTL and other malignancies should be considered in indeterminate ALF, even without imaging findings, atypical lymphocytes in peripheral blood/ascites, or overt clinical suspicion of malignancy.

## Consent

Written informed consent was not obtained due to the patient’s death prior to manuscript preparation. The case is presented in an anonymized manner, with no identifiable personal data.

## Conflicts of Interest

The authors declare no conflicts of interest.

## Funding

No funding was received for this manuscript.
